# Small field detector correction factors: effects of the flattening filter for Elekta and Varian linear accelerators

**DOI:** 10.1120/jacmp.v17i3.6059

**Published:** 2016-05-08

**Authors:** Madelaine K. Tyler, Paul Z.Y. Liu, Christopher Lee, David R. McKenzie, Natalka Suchowerska

**Affiliations:** ^1^ Shoalhaven Cancer Care Centre Nowra NSW Australia; ^2^ Department of Medical Physics Prince of Wales Hospital Sydney Australia; ^3^ School of Physics, University of Sydney Australia; ^4^ Chris O'Brien Lifehouse Sydney Australia; ^5^ Central Coast Cancer Centre, Gosford Hospital Gosford Australia

**Keywords:** flattening filter‐free, small‐field dosimetry, correction factors

## Abstract

Flattening filter‐free (FFF) beams are becoming the preferred beam type for stereotactic radiosurgery (SRS) and stereotactic ablative radiation therapy (SABR), as they enable an increase in dose rate and a decrease in treatment time. This work assesses the effects of the flattening filter on small field output factors for 6 MV beams generated by both Elekta and Varian linear accelerators, and determines differences between detector response in flattened (FF) and FFF beams. Relative output factors were measured with a range of detectors (diodes, ionization chambers, radiochromic film, and microDiamond) and referenced to the relative output factors measured with an air core fiber optic dosimeter (FOD), a scintillation dosimeter developed at Chris O'Brien Lifehouse, Sydney. Small field correction factors were generated for both FF and FFF beams. Diode measured detector response was compared with a recently published mathematical relation to predict diode response corrections in small fields. The effect of flattening filter removal on detector response was quantified using a ratio of relative detector responses in FFF and FF fields for the same field size. The removal of the flattening filter was found to have a small but measurable effect on ionization chamber response with maximum deviations of less than ±0.9% across all field sizes measured. Solid‐state detectors showed an increased dependence on the flattening filter of up to ±1.6%. Measured diode response was within ±1.1% of the published mathematical relation for all fields up to 30 mm, independent of linac type and presence or absence of a flattening filter. For 6 MV beams, detector correction factors between FFF and FF beams are interchangeable for a linac between FF and FFF modes, providing that an additional uncertainty of up to ±1.6% is accepted.

PACS number(s): 87.55.km, 87.56.bd, 87.56.Da

## I. INTRODUCTION

Conventional medical linear accelerator (linac) design incorporates a conical flattening filter (FF) in the beam line to create a beam with a uniform intensity at a specified depth in a patient. In small fields, the value of a flattening filter is limited.^(1)^ Recently, linac manufacturers have introduced flattening filter‐free (FFF) beams, which offer the clinical benefit of a higher dose rate with shorter treatment times.^(2)^

**Figure 1:**
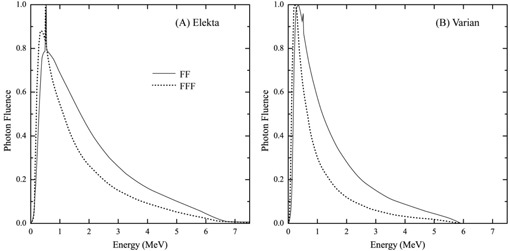
Energy spectra of Elekta and Varian 6 MV FFF and FF beams on the beam central axis, adapted from Javedan et al.^(3)^ and Dalaryd et al.^(4)^ for a 20 × 20 cm and 40 × 40 cm field size respectively.

To create the FFF beam, both Elekta and Varian linacs have the flattening filter replaced with a thin plate of medium‐Z material, inserted between the target and the monitor chamber, to minimize electron contamination and to ensure reproducibility of the monitor chamber response. However, the method used by each vendor to subsequently tune the FFF beam for clinical use differs.^(2)^ The FFF beam for Varian linacs has a softer energy spectrum due to an absence of beam hardening created by the flattening filter. Elekta performs the additional step of effectively ‘matching’ the beam energies by tuning the FFF beam to achieve a match of the percentage depth dose (PDD) of both beams to within 1% at a depth of 10 cm for a 100 mm field. Consequently, the FFF beam spectra for the Elekta and Varian linacs, as seen in Fig. 1, differ from each other and from the respective FF beams, as reported by Javedan et al.^(3)^ and Dalaryd et al.^(4)^ The mean energy on the central axis for a nominal 6 MV photon beam has also been reported by these authors as 1.65 MeV and 1.96 MeV, respectively, for FFF and FF beams generated by an Elekta4 linac and 0.8 MeV and 1.3 MeV respectively for FFF and FF beams generated by a Varian3 linac. Beam characteristics of FFF fields for Elekta and Varian linacs have been previously reported by Foster et al.^(5)^ and Paynter et al.^(6)^

Several studies have reported on the response of ionization chambers, diodes, and the microdiamond^7^, ^8^, ^9^, ^10^, ^11^, ^12^, ^13^, ^14^, ^15^, ^16^, ^17^ detector in small radiation fields for flattened beams, using either experiment or Monte Carlo simulations. Where necessary, small field correction factors $\left(k_{Q_{clin}Q_{msr}}^{f_{clin},f_{msr}}\right)$, as defined by Alfonso et al.,^(18)^ have been provided. These correction factors account for detector over‐ or under response compared to a water‐equivalent detector. This is the first study comparing the effects of removing the flattening filter on a detector output ratio and on the Alfonso correction factors for both Elekta and Varian linac beams.

We compare the output ratios with and without the flattening filter. We hypothesize that changes in the beam characteristics resulting from removal of the flattening filter will cause small, but measurable, changes in the response of solid‐state detectors. Small‐field correction factors for both Elekta and Varian FF and FFF beams for a range of detectors in fields as small as 5 mm are provided.

## II. MATERIALS AND METHODS

A range of commercial and emerging detectors were used to measure relative output factors of 6 MV FF and FFF beams. An Elekta Axesse linear accelerator with Agility MLC (Elekta, Crawley, UK) was used with square MLC fields of nominal widths from 10–100 mm. A Varian iX linear accelerator with Millennium MLC (Varian Medical Systems, Palo Alto, CA) was used to generate square MLC fields of nominal widths from 5–100 mm.

Detectors used for this study included an air core fiber optic dosimeter (FOD) (a scintillation dosimeter designed and manufactured at Chris O'Brien Lifehouse, Australia), Gafchromic EBT3 film (International Specialty Products, Wayne, NJ), a PTW 60019 microDiamond detector (PTW–Frieburg, Germany), an IBA Stereotactic Field Diode (SFD) (IBA Dosimetry, Nuremberg, Germany), a 40 mm^3^ cc04 ionization chamber, and a 10 mm^3^ cc01 ionization chamber (IBA Dosimetry). This selection covers the broad range of detector types in clinical use. The dimensions of the active volume of all real‐time detectors used in this study are provided in Table 1.

The design and performance characteristics of the air core FOD has been previously described in the literature.^19^, ^20^ The air core FOD is a cylindrical scintillation dosimeter with a sensitive volume of 1 mm diameter and 1 mm length, manufactured for use in external beam dosimetry, has been shown to be dosimetrically water‐equivalent, have no directional dependence, and have sufficient spatial resolution for use in field sizes as small as 4 mm in diameter.^10^, ^19^, ^21^ Scintillation dosimeters are a good choice as a reference detector for small field work^15^, ^22^ as their response has been shown to be independent of dose rate, energy, and temperature, and they provide a linear response with delivered dose.^23^, ^24^ Scintillation dosimeters have been benchmarked against film^10^, ^21^ and Monte Carlo^(25)^ and have a smaller measurement uncertainty than film.

Gafchromic film is nearly tissue‐equivalent^(26)^ and can be used as a radiation detector with high spatial resolution. In this study, all films were scanned with an EPSON 10000XL flatbed scanner (SEIKO Epson Co, Hino, Japan). Alfonso et al.^(18)^ recommends radiochromic film for determination of small field correction factors. Although dosimetrically water‐equivalent and suitable for measurement of all field sizes in this study, EBT3 was not used as a reference detector due to the inability to perform real‐time readout and to the larger uncertainty in measurement compared with the FOD.^10^, ^21^ The film was, however, used to validate the accuracy of the FOD relative output factors in the radiation beams investigated.

All measurements (except those with EBT3 film) were performed in a Blue Phantom three‐dimensional scanning water tank (IBA Dosimetry). The effective point of measurement of each detector was placed at a depth of 10 cm in water, using 100 cm source‐to‐chamber distance (90 cm SSD). Each detector was positioned at the center of the radiation field by moving the motorized scanning arm in 0.2 mm increments in both cross‐plane and in‐plane directions to find the position of maximum signal. Hysteresis in the scanning arm movement was minimized by moving the detector in the same direction for all measurements. The diode, microdiamond, and ionization chambers, were oriented with their stems parallel to the radiation beam axis. The air core FOD was oriented with its stem perpendicular to the beam axis to avoid stem effects.

**Table 1:** Summary of active volume dimensions for all real‐time detectors used in this study.

	*Active Volume Dimensions*		
*Detector Type*	*Volume (mm^3^)*	*Diameter (mm)*	*Length (mm)*
Air‐core FOD scintillator	0.79	1.0	1.00
IBA SFD diode	0.017	0.6	0.06
PTW 60019 microDiamond	0.004	2.2	0.001
IBA cc04 ion chamber	40.0	4.0	3.60
IBA cc01 ion chamber	10.0	2.0	3.60

Gafchromic EBT3 film was cut into $50\times 50\;{\text{mm}}^{2}$ pieces and placed between slabs of Gammex Solid Water Type 457 – 310 (Gammex Inc., Middleton, WI) for irradiation. Film processing and analysis followed methods previously described by Tyler et al.^(21)^ For each detector a single measurement session involved collection of three consecutive readings, with the average detector reading calculated. Three independent measurement sessions were performed, with a new setup prior to each session (n=9).

For the smallest fields, volume averaging correction factors were derived, using the methodology described by Ralston et al.,^(10)^ and subsequently applied to detector readings. Volume averaging occurs when the dimensions of the detector perpendicular to the beam central axis are large compared to the uniform region of the radiation beam, which can cause a decrease in the measured signal.^(27)^

Type A uncertainties include detector reproducibility, measurement repeatability, and variation in beam output and measurement setup.^(28)^ Type A uncertainties for measurements with each detector were calculated and reported as a single standard deviation (1 SD) based on multiple measurements, both within a single measurement session and between independent measurement sessions.

### A. Relative output factor measurement

Relative output factors were calculated by normalization of the air core FOD (water‐equivalent detector) reading for a nominal field size, to those of the 30 mm square field size. The 30 mm field size was chosen for two reasons: a) lateral charge particle equilibrium exists for 6 MV beams in water at this field size, and b) some detectors specifically designed for use in small fields have an upper limit on field width (typically 100 mm) due to energy response limitations.

### B. Detector response ratio relative to FOD

A ratio of the relative output factor measured with each detector to the relative output factor measured with the FOD, labeled the response ratio, was determined. Small‐field correction factors were calculated for each detector by evaluating the reciprocal of the response ratio, taking into account detector volume averaging for each field. Correction factors, not incorporating volume‐averaging correction (per the Alfonso et al.^(18)^ formalism) were also calculated.

### C. Changes in detector response between FFF and FF fields

The change in detector response between FFF and FF fields was quantified by dividing the detector response ratio in an FFF beam with the response ratio for the detector in an FF beam for the same field size. The FFF:FF ratio was used to assess the effect of the flattening filter on detector response as a function of field size and linac type.

## III. RESULTS

### A. Relative output factors

**Figure 2:**
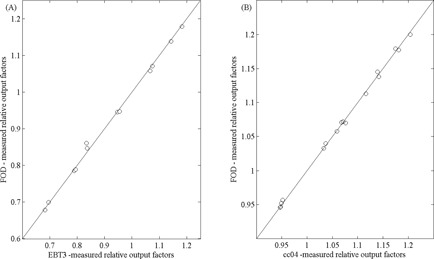
Correlation between FOD measured relative output factors and those measured by (a) EBT3 film for all field sizes and (b) cc04 ionization chamber for fields larger than 10 mm. Measurements from both Elekta and Varian linacs and both FF and FFF beams are included. The solid line represents the line y=x.

The relative output factors measured with the air core FOD and with the EBT3 film (presented in Table 2) agreed to within ±1.1% for all field sizes with correlation shown in Fig. 2(a). FOD‐measured output factors were within ±0.5% of those measured with the cc04 ionization chamber, in fields larger than 10 mm, where spatial (volume) averaging effects are minimal. The cc04 chamber is recommended only for use in fields larger than 10 mm due to the large perturbation and volume averaging corrections required.^(7)^

**Figure 3:**
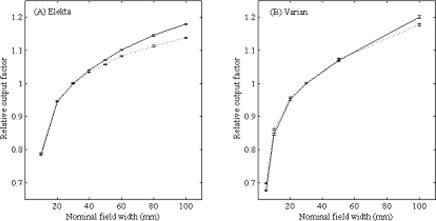
Relative output factors measured with the FOD in FF (solid line) and FFF (dotted line) beams for (a) Elekta and (b) Varian linear accelerators. Relative output factors were measured for field sizes up to 100 mm, referenced to the 30 mm nominal field width.

In fields larger than 10 mm, FOD and cc04 measured output factors were strongly correlated with a correlation coefficient of 0.999 and an RMS deviation of 0.3% for both linacs and beam types, as shown in Fig. 2(b). For fields smaller than 10 mm, EBT3 film is a better choice than the ionization chamber due to its higher spatial resolution and water‐equivalence, although the uncertainty in measurement can be large.^(29)^ The FOD and EBT3 measured output factors had a correlation coefficient of 0.998 and an RMS deviation of 1.1% for all field sizes, as shown in Fig. 2(a). The data in Fig. 3 validate the use of the air core FOD as a reference across a broad range of field sizes.

**Table 2:** FOD and EBT3 measured output factors for nominal field sizes less than 30 mm.

		*Elekta*		*Varian*	
		*Nominal Field Width (mm)*			
*Modality*	*Detector*	*10*	*20*	*5*	*10*
FF	FOD	0.789±0.001	0.946±0.001	0.670±0.002	0.847±0.003
	EBT3	0.789±0.019	0.947±0.023	0.683±0.021	0.837±0.030
FFF	FOD	0.793±0.002	0.947±0.002	0.694±0.002	0.861±0.003
	EBT3	0.794±0.021	0.955±0.019	0.696±0.025	0.834±0.028

Figure 3 shows the relative output factors measured using the air core FOD for FFF and FF beams generated by both Elekta and Varian linacs. The relative output factors measured in FFF fields were less dependent on field size than the corresponding output factors for flattened fields for both Elekta and Varian linacs. This is expected, as one of the major effects of removing the flattening filter is a reduction in head scatter.^(2)^ The Elekta beam shows most of the effects of removing the flattening filter in large fields, while the Varian beam shows effects in both small and large fields.

### B. Detector response ratio relative to FOD

**Figure 4:**
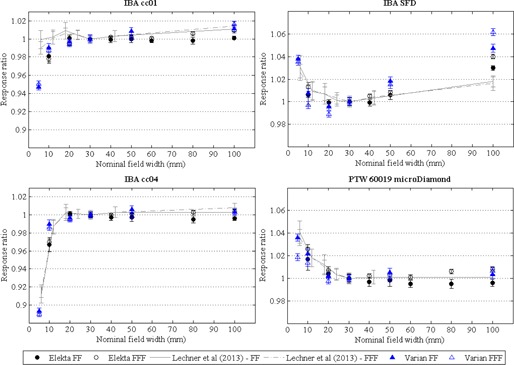
Detector response ratios relative to FOD for air‐filled ionization chambers (IBA cc01 and IBA cc04) and solid‐state detectors (IBA SFD and PTW microDiamond) measured in Elekta and Varian FF and FFF fields for nominal field widths up to 100 mm (referenced to the 30 mm nominal field). Error bars represent Type A uncertainties of ±1 SD. Dose response ratios from Lechner et al.^(16)^ are shown for comparison.

Detector response ratios, relative to the air core FOD, measured for each detector in FF and FFF fields with nominal field sizes up to 100 mm are shown in Fig. 4. Previously published data from Lechner et al.^(16)^ who used alanine as the reference detector, are shown for comparison. An accurate quantitative comparison of the response ratios between the studies was not possible due to the different nominal field sizes used as a result of differences in MLC design.

The SFD diode detector exhibited an overresponse in fields less than 20 mm, with a maximum overresponse of 1.3% (for a 10 mm field) on the Elekta linac and 3.8% (for a 5 mm field) on the Varian linac. The microDiamond also exhibited an overresponse in small fields, reaching a maximum of 2.6% at the smallest field (10 mm) on the Elekta linac and 3.6% at the smallest field (5 mm) on the Varian linac.

Air‐filled ionization chambers had detector response ratios of less than unity (an underresponse) for all field sizes less than 20 mm, despite being corrected for volume averaging. For field sizes greater than 10 mm, ionization chamber relative output factors were within ±0.6% of FOD measurements. The measured detector response ratios are consistent with those published by Lechner et al.^(16)^ across all field sizes.

Type A uncertainties (1 SD) calculated for FF and FFF fields, both within and between independent measurement sessions, were less than ±0.6% for FOD and ±3.6% for EBT3 measurements. Uncertainties for the cc04 and cc01 ionization chambers were within ±0.4% across all field sizes in both FF and FFF fields. The uncertainties for the microDiamond and the SFD were within ±0.4% and ±0.5%, respectively.

Small field correction factors, with and without compensation for volume averaging effects are presented in Appendix A and B, respectively, for Elekta and Varian FF and FFF beams.

### C. Changes in detector response between FFF and FF fields

**Figure 5:**
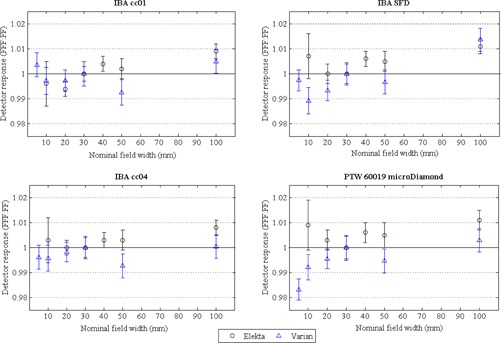
Ratio of detector response in a FFF field to the response in a FF field as a function of nominal field width, referenced to the 30 mm nominal field width for air‐filled ionization chambers and solid‐state detectors used in this study. A horizontal line at 1 is included to illustrate detector response independent of a flattening filter at all field sizes. Type A uncertainties are reported as ±1 SD.

The FFF:FF ratio for air‐filled ionization chambers and solid‐state detectors are shown in Fig. 5. Ionization chamber responses ratios were within ±1.0% (ratio of 0.99–1.01) for all fields investigated, independent of linac vendor. Solid‐state detectors showed an increased dependence on beam generation type with ratios showing up to ±1.6% deviation from unity.

## IV. DISCUSSION

The aim of this study was to compare small field output factors for 6 MV beams generated by Elekta and Varian linear accelerators with and without a flattening filter. Differences in the response of a range of detectors in these fields were determined. A practical outcome of the study is to answer the question as to whether different Alfonso small field corrections are needed when the flattening filter is removed.

The small field response of the solid‐state detectors, the IBA SFD, and PTW microDiamond (Fig. 5), changes when the flattening filter is removed, which requires explanation. We consider two contributions to the detector signal: one originating from the target (focal) and one originating from scatter in the linear accelerator treatment head, including the flattening filter (extra‐focal). Published Monte Carlo simulations have determined the proportion of focal and extra‐focal photons contributing to the dose measured on the central axis as a function of field size for a Varian linear accelerator fitted with a flattening filter.^(30)^ In small fields, the contribution of extra‐focal photons was reported to be minimal, with focal photons dominant. The dose from focal photons was independent of field size, down to a 12 mm field. When the field was reduced further, source occlusion was the main reason for loss of signal. Hug et al.^(30)^ reported the contribution to dose from the flattening filter to be 0.5% for a 30 mm field, a finding consistent with Ding^(31)^ who reported a dose contribution from the flattening filter for a Varian linac of 0.9%–3.0% for 40 mm and 400 mm field sizes, respectively. As a consequence, photons originating in the flattening filter itself are not likely to be the cause of changes in the detector response in small fields; however, this does not preclude changes arising from the absorption of low‐energy components of the beam by the presence of the flattening filter. Detectors with mass‐energy absorption coefficients different to that of water are known to exhibit photon energy dependence in their response^(32)^ that can lead to an overresponse in large fields as a result of the presence of low‐energy photons.^33^, ^34^ In particular, unshielded silicon diodes are known to overrespond in large fields due to the mass density of silicon being different from water.^(32)^ The presence of a steel central electrode in the cc01 chamber is also known to lead to an overresponse in large fields.^35^, ^36^ In small radiation fields, the subject of this study, the presence of low‐energy photons is limited and the energy dependence of the SFD and cc01 ion chamber is expected to be negligible. The microDiamond is also expected to have a small energy dependence as the mass‐energy absorption coefficient of diamond is similar to that of water. This has been confirmed by Laub and Crilly^(37)^ in MV photon beams. As a result, the effects of photon energy change are not expected to contribute to the deviation in the microDiamond detector response between FFF and FF modes seen in Fig. 5.

The overresponse in small radiation fields of silicon diodes, which incorporate silicon of density 2.3 g/cm^3^ and possibly other high‐density material in their sensitive volumes and surroundings, has been well documented.^7^, ^8^, ^9^, ^10^, ^11^, ^12^, ^13^, ^14^, ^15^, ^16^, ^17^, ^38^, ^39^, ^40^, ^41^ Diamond detectors, which have a sensitive volume of density 3.5 g/cm^3^, have been reported to overrespond in small fields.^16^, ^39^, ^41^, ^42^ Monte Carlo simulations have shown that the magnitude of overresponse is proportional to the mass density of the material in the volume of interest, with a larger overresponse determined for a voxel of the same size in a radiation beam for diamond than for silicon in a 5 mm field for 15 MV.^(38)^ Similarly, air‐filled ionization chambers will show an underresponse compared to water, due to the low density of air compared with water in the sensitive volume. Volume‐averaging corrected detector output ratios (Fig. 4) demonstrate the effect of detector density in small fields for all detectors — solid‐state detectors overrespond and air‐filled ionization chambers underrespond.

The density effect is present in both FF and FFF fields. However, there is limited information in the literature on the effect of beam energy on the overresponse of solid‐state detectors in small radiation fields. Cranmer‐Sargison et al.^(11)^ investigated the effect of source parameterization changing incident beam energy over a small range (5.5–6.5 MeV) on diode small field correction factors using Monte Carlo simulations, finding marginal changes in correction factors across such small energy changes. Despite this, we hypothesize that the density effect in small radiation fields is dependent on beam energy and is the source of deviations in FFF:FF ratios for the Varian linac, as presented in Fig. 5. This effect is not as pronounced for Elekta beams due to the smaller difference in mean energy between FFF and FF modes. Monte Carlo simulations are needed to characterize the effect of beam energy on the response of these high‐density detectors to enable a definitive answer. Our measured data agree with results presented by Lechner et al.^(16)^ for Elekta linacs where there is minimal deviation in detector measured output factors between FFF and FF fields. Underwood et al.^(43)^ determined a similar deviation in small field correction factors for FFF and FF fields for a diode and the PTW microDiamond detector for a Varian linac.

Results from this study indicate that, with the implementation of FFF beams, there is a small but clinically insignificant effect on measured small field correction factors. Detector correction factors may be used interchangeably between FF and FFF beams for a 6 MV beam, though an additional uncertainty of up to ±1.6% is introduced.

**Figure 6:**
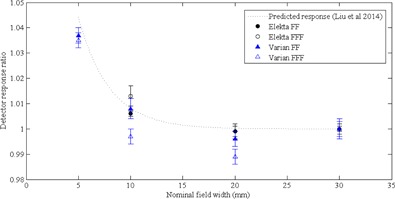
Predicted (Liu et al.^(44)^: dotted line) and measured detector response for the SFD in radiation fields up to 30 mm. Type A uncertainties are reported as ±1 SD.

Recently, Liu et al.^(44)^ published a mathematical relation to predict detector response of diodes in FF beams. The mathematical relation was based on multiple experimental measurements in FF fields using SFD diodes in beams generated by Siemens, Elekta, and Varian linear accelerators and can be used to predict diode overresponse in small fields, independent of linac type. Detector response ratios calculated in this study were compared with those predicted by Liu and colleagues and are shown in Fig. 6. Measured FF detector response ratios were within ±0.7% of the predicted response by the Liu study, indicating that the model can be extended to predict detector response and small field correction factors for the SFD response in FF beams. In FFF beams, the measured detector response ratios showed a larger deviation (± 1.1%) from the predicted ratio, which is expected as the mathematical relation was built on FF data. The prediction of correction factors for FFF beams is still possible, provided the additional uncertainty is acceptable. A similar mathematical relation for the overresponse of the PTW microDiamond in small fields could also be developed.

Kamio and Bouchard^(45)^ have suggested implementation of ‘limits of usability’ for individual detectors in small fields, describing the range of field sizes where detector perturbations are small and do not require direct measurement of correction factors. In situations where the highest accuracy is required, it is recommended to measure correction factors specific for the linear accelerator for each detector with a suitable reference detector (i.e., Gafchromic film, scintillators or Alanine) or by Monte Carlo simulations. If ±5% accuracy in the measurement of output factors is deemed acceptable, Alfonso small field correction factors may be used interchangeably in fields as small as 5 mm between FFF and FF modes for both Elekta and Varian linacs. Furthermore, in the absence of a suitable reference detector, we have confirmed that the universal SFD diode correction factors of Liu et al.^(44)^ can be used to predict detector response with an accuracy of ±1.1%.

## V. CONCLUSIONS

Small field output factors were measured for a range of detectors in FFF and FF fields on both Elekta and Varian linear accelerators. It is known that FFF beams are generated differently on Elekta and Varian linear accelerators, with energy ‘matching’ performed on Elekta linacs to increase the energy of the FFF beam to match the penetration of the FF beam 10 cm depth for a 100 mm square field. The Varian FFF beam is not matched and consequently has a much softer spectrum than the FF mode.

We have demonstrated that output factors (and small field correction factors) for a given field size remain within ±1.0% for ionization chambers between FFF and FF modalities on both Elekta and Varian linacs. A larger deviation between correction factors for FF and FFF modalities of up to ±1.2% and ±1.7% for Elekta and Varian linacs, respectively, was observed for solid‐state detector. The largest deviations between FF and FFF modalities were observed in the smallest fields for Varian beams. Deviations in detector small field correction factors between FF and FFF beams are sufficiently small (up to a maximum of ±1.7%) to indicate the potential interchangeability of correction factors between FF and FFF fields on the same linac.

The mathematical relation developed for the IBA SFD diode in FF fields was found to be accurate in predicting SFD response to within ±0.6% in FF fields and ±1.1% in FFF fields. A similar mathematical relation could be developed for the PTW microDiamond for the calculation of small field correction factors.

## ACKNOWLEDGMENTS

The authors acknowledge Anna Ralston for valuable input into scientific discussions, Kirbie Sloan for her support in data collection, and Peter Douglas, Nucletron Australia, for the loan of the PTW 60019 microDiamond used in this study.

## COPYRIGHT

This work is licensed under a Creative Commons Attribution 4.0 International License.

## Appendix A: Small Field Correction Factors for 6 MV Elekta FF and FFF Fields.

**Table A1:** Detector readings compensated for volume‐averaging.

		*Nominal Field Width (mm)*	
*Detector*	*Delivery Mode*	*10*	*20*
IBA SFD	FF	0.994±0.001	1.001±0.002
	FFF	0.987±0.004	1.001±0.003
PTW 60019 microDiamond	FF	0.983±0.004	0.996±0.002
	FFF	0.974±0.004	0.993±0.003
IBA cc01	FF	1.019±0.001	0.999±0.001
	FFF	1.023±0.003	1.005±0.002
IBA cc04	FF	1.034±0.001	0.999±0.001
	FFF	1.031±0.001	0.999±0.002

Note: Correction factors are dependent on the detector material type and independent of the size of the detector active volume, as a result of the correction for volume averaging. The correction factors presented apply only to the specific detectors and radiation beams used in this study; however, they may serve as a convenient comparison for correction factors measured in local conditions.

**Table A2:** Detector readings not compensated for volume‐averaging.

		*Nominal Field Width (mm)*	
*Detector*	*Delivery Mode*	*10*	*20*
IBA SFD	FF	0.998±0.001	1.002±0.002
	FFF	0.983±0.004	1.001±0.003
PTW 60019 microDiamond	FF	0.986±0.004	0.996±0.002
	FFF	0.969±0.004	0.993±0.003
IBA cc01	FF	1.020±0.001	0.999±0.001
	FFF	1.024±0.003	1.002±0.002
IBA cc04	FF	1.060±0.001	1.006±0.001
	FFF	1.059±0.001	1.004±0.002

Note: Correction factors are dependent on the detector material type and independent of the size of the detector active volume, as a result of the correction for volume averaging. The correction factors presented apply only to the specific detectors and radiation beams used in this study; however, they may serve as a convenient comparison for correction factors measured in local conditions.

## Appendix B. Small Field Correction Factors for 6 MV Varian FF and FFF Fields.

**Table B1:** Detector readings compensated for volume‐averaging.

		*Nominal Field Width (mm)*		
*Detector*	*Delivery Mode*	*5*	*10*	*20*
IBA SFD	FF	0.953±0.005	0.992±0.002	1.003±0.002
	FFF	0.959±0.002	1.003±0.002	1.008±0.003
PTW 60019 microDiamond	FF	0.955±0.003	0.979±0.004	0.998±0.003
	FFF	0.971±0.003	0.989±0.003	1.002±0.003
IBA cc01	FF	1.045±0.003	1.010±0.004	1.003±0.003
	FFF	1.047±0.003	1.013±0.003	1.006±0.003
IBA cc04	FF	1.107±0.003	1.010±0.004	1.003±0.003
	FFF	1.116±0.003	1.010±0.003	1.005±0.003

Note: Correction factors are dependent on the detector material type and independent of the size of the detector active volume, as a result of the correction for volume averaging. The correction factors presented apply only to the specific detectors and radiation beams used in this study; however, they may serve as a convenient comparison for correction factors measured in local conditions.

**Table B2:** Detector readings not compensated for volume‐averaging.

		*Nominal Field Width (mm)*		
*Detector*	*Delivery Mode*	*5*	*10*	*20*
IBA SFD	FF	0.952±0.005	0.992±0.002	1.004±0.002
	FFF	0.958±0.002	1.006±0.002	1.008±0.003
PTW 60019 microDiamond	FF	0.954±0.003	0.976±0.004	0.998±0.003
	FFF	0.970±0.003	0.989±0.003	1.002±0.003
IBA cc01	FF	1.052±0.003	1.010±0.004	1.003±0.003
	FFF	1.061±0.003	1.013±0.003	1.006±0.003
IBA cc04	FF	1.188±0.003	1.027±0.004	1.003±0.003
	FFF	1.200±0.003	1.031±0.003	1.005±0.003

Note: Correction factors are dependent on the detector material type and independent of the size of the detector active volume, as a result of the correction for volume averaging. The correction factors presented apply only to the specific detectors and radiation beams used in this study; however, they may serve as a convenient comparison for correction factors measured in local conditions.
